# Experimental Realisation of High-sensitivity Laboratory X-ray Grating-based Phase-contrast Computed Tomography

**DOI:** 10.1038/srep24022

**Published:** 2016-04-04

**Authors:** Lorenz Birnbacher, Marian Willner, Astrid Velroyen, Mathias Marschner, Alexander Hipp, Jan Meiser, Frieder Koch, Tobias Schröter, Danays Kunka, Jürgen Mohr, Franz Pfeiffer, Julia Herzen

**Affiliations:** 1 Lehrstuhl für Biomedizinische Physik, Physik-Department & Institut für Medizintechnik, Technische Universität München, Garching, Germany; 2 Zentrum für Material- und Küstenforschung, Helmholtz-Zentrum Geesthacht, Geesthacht, Germany; 3 Institut für Mikrostrukturtechnik, Karlsruher Institut für Technologie (KIT), Karlsruhe, Germany

## Abstract

The possibility to perform high-sensitivity X-ray phase-contrast imaging with laboratory grating-based phase-contrast computed tomography (gbPC-CT) setups is of great interest for a broad range of high-resolution biomedical applications. However, achieving high sensitivity with laboratory gbPC-CT setups still poses a challenge because several factors such as the reduced flux, the polychromaticity of the spectrum, and the limited coherence of the X-ray source reduce the performance of laboratory gbPC-CT in comparison to gbPC-CT at synchrotron facilities. In this work, we present our laboratory X-ray Talbot-Lau interferometry setup operating at 40 kVp and describe how we achieve the high sensitivity yet unrivalled by any other laboratory X-ray phase-contrast technique. We provide the angular sensitivity expressed via the minimum resolvable refraction angle both in theory and experiment, and compare our data with other differential phase-contrast setups. Furthermore, we show that the good stability of our high-sensitivity setup allows for tomographic scans, by which even the electron density can be retrieved quantitatively as has been demonstrated in several preclinical studies.

Over the last two decades, it has been shown that phase-contrast imaging provides superior soft-tissue contrast in comparison to conventional attenuation based X-ray imaging^1^,^2^. Among the X-ray phase-contrast imaging methods that are generally mainly available at synchrotron sources grating-based differential phase-contrast (DPC) imaging can also be realised with incoherent laboratory X-ray sources^3^,^4^. This essential development increases the availability of phase-contrast imaging, and allows for a wide range of biomedical applications. Moreover, it is possible to perform grating-based phase-contrast computed tomography (gbPC-CT) in a quantitative manner with this technique by retrieving the electron density^5^,^6^. However in comparison to synchrotron facilities, the performance of laboratory phase-contrast setups is usually compromised by the polychromaticity of the X-ray spectrum, the lower spatial resolution due to a larger source size, the incoherence of the radiation, and the limited flux.

One of the current challenges for laboratory phase-contrast imaging is the need for setups that are able to detect extremely small refraction angles, i.e. setups that exhibit high sensitivity in combination with high spatial resolution. The sensitivity has already been thoroughly investigated^7^,^8^,^9^: the angular sensitivity was introduced in literature as the smallest detectable refraction angle depending on the setup geometry and the noise behaviour in the DPC projections. In order to boost the sensitivity, the inter-grating distance between the phase and the analyser grating has to be increased, and the period of the analyser grating should be as small as possible.

Additionally, high visibility of the analysed interference patterns and thus an optimised performance of the interferometer are needed to achieve high sensitivity. Especially the design energy of the setup depending on the X-ray spectrum, the grating duty-cycle, and the inter-grating distances have to be chosen carefully. The choice and optimisation of the geometry of the setup have already been investigated by Donath *et al.* and Engelhardt *et al.*^10^,^11^ concluding that it is advantageous to position the sample as close as possible to the phase grating. Moreover, one can select the design energy independently from the inter-grating distances enabling a large variety of DPC setups for different applications^4^,^6^,^12^. The spatial resolution is also a critical factor to keep in mind as high sensitivity can be achieved while at the same time the spatial resolution may be unsuited for the desired application.

First results of tomographic scans with a laboratory setup have been described by Weitkamp *et al.*^13^ and Pfeiffer *et al.*^14^, and studies on quantitative imaging showed that the use of a water container surrounding the sample significantly improves the imaging results due to reduction of phase-wrapping and beam hardening artefacts^5^,^15^,^16^.

Recently, new developments in grating fabrication^17^,^18^, the theoretical and experimental optimisation of the setup design^12^,^19^, and more advanced processing algorithms^20^ have lead to a substantial rise in quality and stability of DPC imaging, namely in an increase of interferometric visibility and a reduction of noise.

In this work, we describe the design of a gbPC-CT setup by refining the aforementioned results with additional measures to reach high sensitivity for tomographic scans, which mainly depends on reaching high sensitivity in DPC projections. Further, we determined the angular sensitivity to provide a benchmark for comparison to synchrotron facilities and other laboratory PC-CT setups. As a final result, we show an exemplary tomographic scan of a biomedical specimen visualizing slight differences in the refractive index decrement with a sensitivity comparable to results from synchrotrons, and – in combination with energy calibration – the resolvable electron density resolution.

## Angular Sensitivity

**Figure 1:**
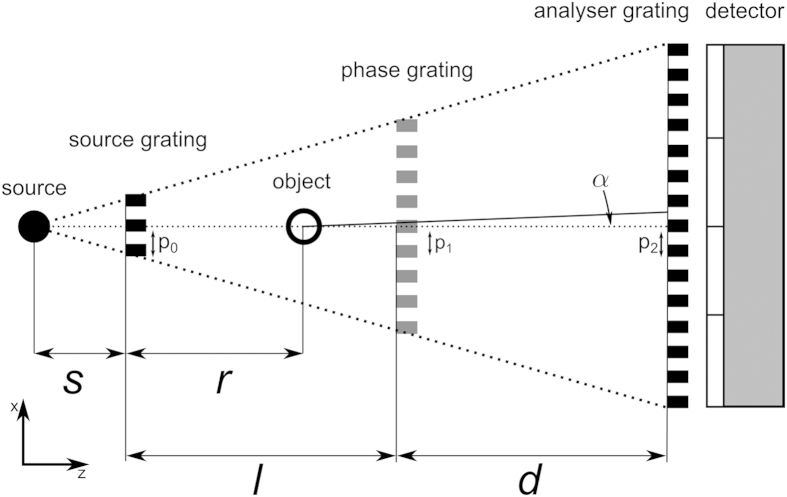
Schematic of a gbPC-CT setup. From left to right: the X-ray source, the three gratings source, phase, and analyser grating with their corresponding periods *p*_0_, *p*_1_, and *p*_2_ followed by the detector. The source grating is placed at distance *s* away from the source and the object is placed at distance *r* away from the source grating inducing a refraction angle of *α*, which is increased by a factor (*l* + *s*)/(*r* + *s*) taking into account the magnification of *α* by the distance from the object to the phase grating^11^. The figure is not to scale as in particular the refractive angle and grating periods are much smaller.

A laboratory gbPC-CT setup (or X-ray Talbot-Lau interferometer) consists of an X-ray source, an X-ray detector, and three gratings (cf. Fig. 1). The first grating, the so-called source grating, enables the use of incoherent X-ray sources creating an array of partially coherent line sources^4^. The second grating, the phase grating, works as a beam splitter and creates a periodic re-appearance of the interference pattern due to the Talbot effect. In order to resolve this interference pattern, an analyser grating is used because the period of the interference pattern is usually smaller than the detector pixel pitch. We refer to other publications describing how the attenuation, differential-phase, and dark-field signal can be extracted from the measured intensity signal^13^,^21^.

An object placed into the beam of a Talbot-Lau interferometer induces an inclination of the X-ray wave front by an angle *α*, which can be detected measuring the lateral shift of the interference pattern *φ*. Thereby, the resulting refraction angle can be expressed as


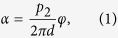


with *d* being the distance between the phase and the analyser grating, and *p*_2_ being the period of the analyser grating (cf. Fig. 1). We use the definition of the angular sensitivity as the minimum resolvable refraction angle^9^


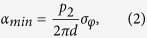


with *p*_2_ and *d* forming the term depending on the setup geometry, and *σ*_*φ*_ being the standard deviation (or noise) of *φ*. With a single-photon counting detector, the noise in the DPC projections can be directly expressed as^9^,^22^


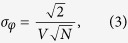


depending on the interferometric visibility *V*, which describes the quality of the interferometer, and the total number of counts *N*.

Since DPC projections require a second image without sample in the beam for reference correction, an additional factor of 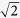 has to be considered^8^. As the sample is not positioned directly at the position of the phase grating, the measured refraction angle has to be multiplied with a factor (*l* + *s*)/(*r* + *s*) with *s* being the distance from the source to the source grating, *r* being the distance from the source grating to the object, and *l* being the distance between the source grating and the phase grating (cf. Fig. 1)^11^. Taking those aspects into account, the resulting formula for the angular sensitivity in a DPC projection is


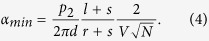


The phase-shift and thus the refraction angle are proportional to the refractive index decrement *δ*, which is linked to the electron density *ρ*_*e*_ by


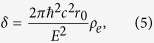


where *r*_0_ is the classical electron radius, *ħ* is the reduced Planck constant, *c* is the speed of light, and *E* is the X-ray energy.

As it is obvious from the geometry dependent factor in Eq. (2), the angular sensitivity can be increased using a smaller grating period *p*_2_, which is limited by the currently available grating fabrication technique. Moreover, *α*_*min*_ can be optimised by increasing the inter-grating distance *d*. However, the benefit of this would be largely compensated by the loss in flux while retaining the same exposure time. Also, a small change in the inter-grating distance can have large impact on the interferometric visibility at small periods and a polychromatic source^12^. Alternatively, a source with a higher power output would allow to increase *d*. Furthermore, a higher visibility due to improved grating quality increases the overall achievable sensitivity. Increasing the exposure time and thus the number of photons would also increase the sensitivity or reduce the minimum resolvable refraction angle, respectively. Although there is no theoretical limit in Eq. (4) for reaching high sensitivity, setup dependent instabilities like vibrations and thermal fluctuations represent an experimental boundary.

When a tomographic scan is performed, additional parameters influence the achievable resolution of the refractive index decrement *σ*_*δ*_, such as the sampling rate depending on the number of projections and the filter choice of the filtered backprojection, which has been investigated elsewhere^22^,^23^,^24^. Furthermore, it is possible to retrieve the electron density resolution in a sample using a mean energy for the polychromatic spectrum^5^. The electron density resolution 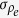 can be calculated reformulating Eq. (5) into


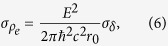


from which the strong energy dependence of the electron density resolution gets apparent.

## Setup Design for High Angular Sensitivity

**Figure 2:**
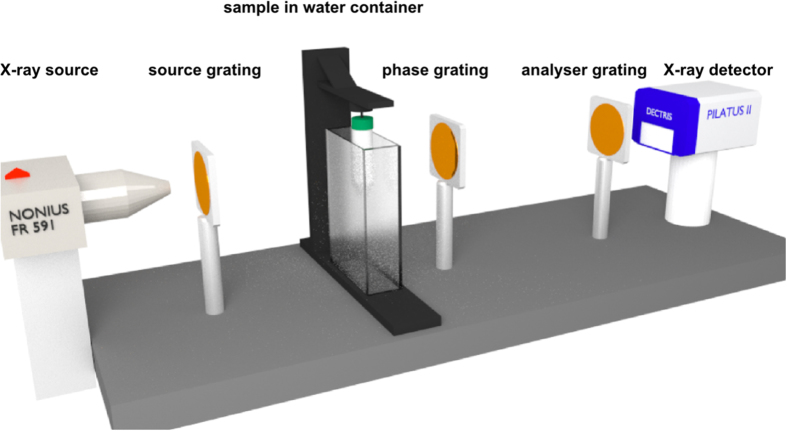
Sketch of the experimental gbPC-CT setup. From left to right: the rotating anode X-ray source followed by the source grating. The next element in the beam is the water container and the sample stage for tomography. Directly behind the sample is the phase grating. The analyser grating and the Pilatus II detector are installed at a discrete distance behind the phase grating. Figure adapted from Willner *et al.*^43^.

In order to realise experimentally a very sensitive DPC setup for a design energy of 27 keV, we use an Enraf Nonius FR 591 rotating anode with a molybdenum target as X-ray source operating at 40 kVp and 70 mA (cf. Fig. 2). The rotating anode with the molybdenum spectrum provides reasonably high flux at this energy range. As a detector, we use a Dectris Pilatus II 100 k single-photon counting detector with a 1 mm thick silicon sensor and 487 × 195 pixels. The pixel pitch is 172 × 172 μm^2^. Besides the already mentioned single-photon counting property, the lack of readout noise and the box-like point spread function are the main advantages of this type of detector^25^. Major limitations are the poor quantum efficiency at higher energies (approx. 20% at 35 keV for silicon) and the rather large pixel size.

The inter-grating distances *l* and *d* (cf. Fig. 1) are equal to 85.7 cm. They were optimised to obtain the maximum visibility for the polychromatic spectrum and do not correspond to a discrete Talbot distance^12^. The choice of a symmetric setup and a phase grating that introduces a phase shift of *π* to the wave front is favourable with regard to a better spectral acceptance at higher Talbot orders^12^,^19^. The periods of all gratings were 5.4 μm. Smaller grating periods result in higher aspect ratios, which are more difficult to fabricate technically and thus tend to perform worse than gratings with larger periods. In general, one has to take into account the effect of shadowing by planar grating bars. In our case, the source grating was placed 56 cm away from the X-ray source to reduce the effect of shadowing artefacts, which could be otherwise addressed with bent gratings. The gratings were fabricated on 200 μm thick silicon wafers, which cause a loss in flux due to absorption. The filling height of the source and the analyser gratings were approximately 70 μm, respectively. The setup design energy of 27 keV corresponds to a filling height of 5.2 μm gold of the phase grating for a *π*-shift. The duty cycles of the three gratings are approximately 0.6, which is considered to be optimal on a theoretical basis^19^. All gratings were developed and fabricated by the Institut für Mikrostrukturtechnik, Karlsruher Institut für Technologie (Karlsruhe, Germany).

The spatial resolution is limited by the extended source size, which was determined to be 132 and 226 μm in x- and y-direction (FWHM), respectively. The optimum magnification in x- and y-direction and thus the effective pixel sizes can be calculated to 2.30 and 1.76 or 75 and 98 μm, respectively^26^. Therefore, we chose a sample magnification of 1.72 to get an effective pixel size of 100 × 100 μm^2^, where the influence of the extended source size does not yet have to be taken into account.

As already mentioned, the measured sample was positioned as close as possible to the phase grating to have a high angular sensitivity^10^. Additionally, the sample was immersed into a water container with rectangular profile to avoid phase-wrapping (cf. Fig. 2) and reduce the effect of beam hardening, which improves the quality of tomographic scans and enables quantitative imaging^15^. The electron density can be retrieved quantitatively if the effective energy *E* of the polychromatic spectrum is known. This can be achieved via energy calibration using well-known homogeneous materials in a tomographic scan, e.g. a PMMA (polymethylmethacrylate) rod as used in this setup^5^,^6^,^16^. Without the beam hardening reduction induced by the water container and the calibration in tomographic scans, the effective energy could differ strongly for different materials and render quantitative imaging impossible^27^.

The DPC projections in this study were acquired with 11 phase-steps^13^ and a polynomial fit was used to remove the phase ramp in the DPC signal^20^. The mean visibility was 38.7%.

For the tomographic scan, the exposure time per phase-step was 5 seconds and the flat-field projections without the sample in the beam were taken each 20 projections. The tomographic reconstruction of the 1200 projections for each image signal over a full rotation was performed with a filtered backprojection algorithm using a Hilbert filter for the DPC projections^28^.

The sample was part of a human cerebellum fixed with a 4-%-formalin solution and put in a 15 mL Falcon tube. The sample was excised at the Institut für Rechtsmedizin (Ludwig-Maximilians-Universität München, Germany) and the experiment was approved by the local ethics committee (Projectnumber 319/13, Ethikkommission, Fakultät für Medizin, Technische Universität München, Germany).

## Results

**Figure 3:**
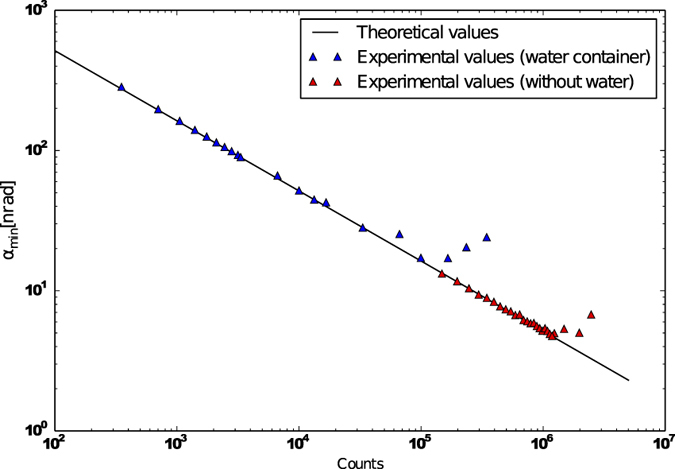
Log-log plot of the minimum resolvable refraction angle *α*_*min*_ depending on the number of counts at a visibility of 38.7%. The blue data points were measured with a water container in the beam. The red data points represent results achieved without a water container in the beam. The standard deviation of the lateral phase-shift *σ*_*φ*_ contributing to *α*_*min*_ according to Eq. (4) was determined in an area of 190 × 190 pixels.

First, we measured DPC projections with the water container in the beam at different exposure times and validated the existing model of the angular sensitivity depending directly on the visibility and the number of photons as formulated in Eq. (4). The measured resulting minimum resolvable refraction angles *α*_*min*_ are represented by the blue triangles in Fig. 3. With increasing number of photons starting at approximately 360 counts, *α*_*min*_ decreases as expected according to the theoretical considerations. At about 1.6 × 10^5^ photons the minimum refraction angle starts to increase again. This can be explained by instabilities due to thermal drift and jitter of the gratings during the relatively long exposure times corresponding to this number of counts^29^. The minimum achievable refraction angle *α*_*min*_ with water container was determined to be 17 nrad at approximately 1.0 × 10^5^ photons corresponding to 275 s exposure time per DPC projection. However, this exposure time was with 275 s per DPC projection due to the 11 steps too long to perform tomographic scans in a reasonable amount of time. Therefore, we chose an exposure time of 55 s per DPC projection for the tomographic scans corresponding to a sensitivity of 38 nrad.

Additionally, we measured the angular sensitivity without the water container. The flux is approximately increased by a factor of 10 (cf. red triangles in Fig. 3). The smallest value of minimum refraction angle was 5 nrad at 1.2 × 10^6^ photons corresponding to 275 s exposure time per DPC projection. At 55 s total exposure time per projection (2.5 × 10^5^ photons) 10 nrad were measured. However, without water container high-quality tomographic scans are not yet possible due to the artefacts described above.

**Figure 4:**
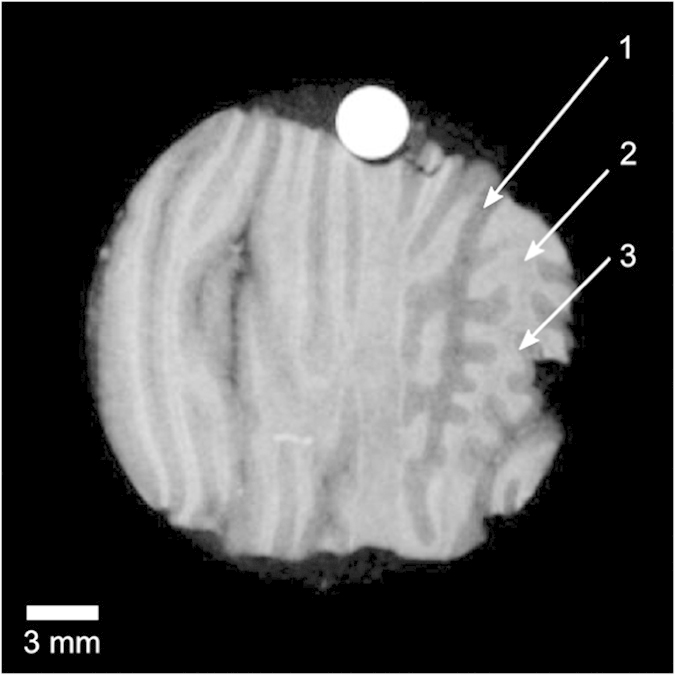
Exemplary tomographic slice of the electron density distribution of a human cerebellum sample measured at 40 kVp. The sensitivity is sufficiently high to reveal subtle differences in the interior structure of the cerebellum: the stratum moleculare (1), the white matter (2), and the stratum granulosum (3). The round PMMA rod (white) is used for energy calibration. The displayed values are in the linear range of 338–356 × 10^27^ electrons/m^3^.

In order to showcase high sensitivity gbPC-CT, an exemplary biomedical application of gbPC-CT, a tomographic scan of a human cerebellum specimen is presented in Fig. 4. Minute differences in soft matter electron density are revealed making it possible to delineate the stratum moleculare from the stratum granulosum and the white matter as illustrated in Fig. 4. To our knowledge, until now this kind of soft-tissue differentiation has been restricted to synchrotron sources^30^,^31^,^32^. Note that the measurement was performed with the laboratory X-ray source described above at a spatial resolution of approx. 170 μm (10% MTF)^33^.

The energy calibration using the mean *δ*-value of the PMMA rod added to the sample (cf. Fig. 4) delivered an effective energy *E* of 25.6 keV and an electron density resolution (standard deviation) of 0.45 × 10^27^ electrons/m^3^ in a volume of 10 × 10 × 10 pixels. The signal-to-noise ratio (SNR) in the same volume was 117 compared to 3 in the attenuation signal.

## Discussion and Conclusion

The best minimum resolvable refraction angle and thus the sensitivity limit reached with this state-of-the-art gbPC-CT setup is 5 nrad with 275 s exposure time for DPC projection without water container and is yet unreported to be achieved by any other laboratory gbPC-CT setup. While this quite long exposure time would find only limited practical application, it shows that high sensitivity gbPC-CT measurements are possible. Also, the sensitivity value of 38 nrad with 55 s exposure time per DPC projection for tomographic scans with water container is unrivalled by other laboratory gbPC-CT setups. An overview of reported sensitivity values is compiled in Table 1 for comparison as no sensitivity limits are given. Note that the referenced setups operate with completely different experimental conditions.

Refs 1 and 2 represent setups installed at a synchrotron source. Ref. 1 is clearly superior in sensitivity and spatial resolution, which can be attributed to the higher flux and the monochromatic performance^30^. Due to geometrical differences our setup is approximately 2.6 times more sensitive than reference setup 2^7^. However, the spatial resolution of reference setup 2 with a pixel size of 7.4 μm is superior. Therefore, increasing the Talbot order or the exposure time, which is quite short in both referenced setups, would most probably deliver higher sensitivity. Generally, DPC imaging at synchrotron facilities outperforms laboratory setups in terms of spatial resolution due to the small source size, and in terms of measurement time due to the high flux. However, the cone beam geometry in laboratory setups provides the advantage of a larger field of view, beneficial for measuring larger samples. And, this study shows that high sensitivity can also be achieved at laboratory setups.

The sensitivity value calculated of data reported in Ref. 3 is approx. 110 nrad^29^ and is based on a measurement performed without a water container and longer exposure time, but higher pixel size. The minimum resolvable refraction angle *α*_*min*_ achieved with our setup is significantly smaller. The main difference grounds on the geometric factor (cf. Eq. (2)), where our setup is 6.8 times more sensitive. The sensitivity values of refs 4 and 5 in Table 1 were achieved with a very compact setup using a low flux microfocus tube that causes the low sensitivity^8^,^9^.

We want to emphasize that we are also able to perform reproducible tomographic scans with the possibility to retrieve quantitative phase-contrast Hounsfield units as demonstrated in several preclinical studies^34^,^35^,^36^,^37^.

A direct comparison to the electron density resolution reported at synchrotron facilities is difficult, as the standard deviation of the refractive index decrement depends on both the measured object and the energy of the experiment. Nonetheless, our value of 0.45 × 10^27^ electrons/m^3^ lies in the range of values calculated from reported measurements at synchrotron facilites (0.1–0.6 × 10^27^ electrons/m^3^)^30^,^31^,^38^,^39^,^40^.

Edge-illumination (coded aperture) is a different high sensitivity phase-contrast technique which can also be realised with laboratory X-ray sources. One reported value of the minimum resolvable refraction angle with this method is 270 nrad^41^. However, it has to be mentioned that the reported value was reached with a non-optimised setup and the total exposure time was only 14 s.

Laboratory grating-based phase-contrast imaging still suffers from several drawbacks, which have to be addressed. One main issue is the long scan time. This parameter can be reduced with improved X-ray sources, a higher quantum efficiency of the detector, thinner grating substrates, and for a tomographic scan the use of iterative reconstruction^42^. Moreover, the spatial resolution is still limited and not sufficient for certain biomedical applications. In contrast to conventional absorption tomography, a stronger geometric magnification requires an asymmetric setup design which lacks performance and flexibility.

Going towards high-energy applications in both the clinical field and materials science, the overall performance and visibility of gbPC-CT are still limited by the available grating area and the realisation of high-aspect-ratio gratings suited for higher energies.

## Additional Information

**How to cite this article**: Birnbacher, L. *et al.* Experimental Realisation of High-sensitivity Laboratory X-ray Grating-based Phase-contrast Computed Tomography. *Sci. Rep.* **6**, 24022; doi: 10.1038/srep24022 (2016).

## Figures and Tables

**Table 1:** Parameter comparison between the gbPC-CT setup presented in this study and other grating-based DPC setups reported in literature.

Setup	Facility	Period p_2_	Inter-grating distance d	Sensitivity	Exposure time	Pixel size
setup without water container	laboratory	5.4 μm	85.7 cm	5 nrad	275 s	172 μm
setup with water container	laboratory	5.4 μm	85.7 cm	38 nrad	55 s	172 μm
reference setup no. 1^30^	synchrotron	2.0 μm	36.1 cm	14 nrad	3.2 s	28 μm
reference setup no. 2^7^	synchrotron	2.4 μm	12.1 cm	67 nrad	2.1 s	7.4 μm
reference setup no. 3^29^	laboratory	3.0 μm	6.9 cm	110 nrad	80.4 s	48 μm
reference setup no. 4^8^	laboratory	2.4 μm	19.5–32.5 cm	270–300 nrad	128 s	48 μm
reference setup no. 5^9^	laboratory	2.4 μm	10 cm	250 nrad	128 s	48 μm
